# Periparturient blood T-lymphocyte PD-1 and CTLA-4 expression as potential predictors of new intramammary infections in dairy cows during early lactation (short communication)

**DOI:** 10.1186/s12917-024-03977-1

**Published:** 2024-04-20

**Authors:** Ana Cláudia Dumont Oliveira, Carolina Menezes Suassuna de Souza, Eduardo Milton Ramos-Sanchez, Soraia Araújo Diniz, Ewerton de Souza Lima, Maiara Garcia Blagitz, Robson Cavalcante Veras, Marcos Bryan Heinemann, Alice Maria Melville Paiva Della Libera, Sarne De Vliegher, Artur Cezar de Carvalho Fernandes, Fernando Nogueira Souza

**Affiliations:** 1 Veterinary Clinical Immunology Research Group, Departamento de Clínica Médica, Faculdade de Medicina Veterinária e Zootecnia, Universidade de São Paulo, São Paulo, 05508-270 Brazil; 2 Programa de Pós-Graduação em Ciência Animal, Centro de Ciências Agrárias, Universidade Federal da Paraíba, Areia, 58397-000 Brazil; 3 Departamento de Ciências Veterinárias, Centro de Ciências Agrárias, Núcleo Aplicado à Produção e Sanidade da Glândula Mamária (NAPROSA), Universidade Federal da Paraíba, Areia, 58397-000 Areia Brazil; 4 Escuela Profesional de Medicina Humana, Facultad de Medicina, Universidad Nacional Toribio Rodríguez de Mendoza de Amazonas, Chachapoyas, 01000 Peru; 5 Departamento de Salud Publica, Facultad de Ciencias de La Salud, Universidad Nacional Toribio Rodriguez de Mendoza de Amazonas, Chachapoyas, 01000 Peru; 6 Curso de Medicina Veterinária, Instituto de Ciências Agrárias, Universidade Federal dos Vales do Jequitinhonha e Mucuri, Unaí, 38610-000 Brazil; 7 Bem-Estar e Produção Animal Sustentável na Fronteira Sul, Programa de Pós-Graduação em Saúde, Universidade Federal da Fronteira Sul, Realeza, 85770-000 Brazil; 8 Departamento de Ciências Farmacêuticas, Centro de Ciências da Saúde, Universidade Federal da Paraíba, 58051-900 João Pessoa, Brasil; 9 Departamento de Medicina Veterinária Preventiva e Saúde Animal, Faculdade de Medicina Veterinária e Zootecnia, Universidade de São Paulo, São Paulo, 05508-270 Brazil; 10 M-team & Mastitis and Milk Quality Research Unit, Department of Internal Medicine, Reproduction and Population Medicine, Faculty of Veterinary Medicine, Ghent University, Salisburylaan 133, Merelbeke, 9820 Belgium; 11 Departamento de Medicina Veterinária, Centro de Ciências Agrárias, Fazenda São Luiz, Universidade Federal de Alagoas, Viçosa, 57700-000 Brazil

**Keywords:** Immune checkpoints, Mastitis, T-cell, Transition period, Dairy cow

## Abstract

**Background:**

The periparturient period in dairy cows is marked by immunosuppression which increases the likelihood of infectious disorders, particularly also mastitis. An in-depth understanding of peripartum leukocyte biology is vital for the implementation of highly successful post-partum disease prevention measures. Immune checkpoint molecules, such as programmed death 1 (PD-1) and cytotoxic T-lymphocyte antigen 4 (CTLA-4), are critical inhibitory receptors expressed on immune cells, particularly T cells, that drive immunosuppressive signaling pathways. However, the potential role of immune checkpoint molecules expression in T-cells on udder health has never been explored. Thus, the association between the occurrence of new postpartum intramammary infections (IMIs) and the expression of programmed cell death protein 1 (PD-1) and cytotoxic T-lymphocyte-associated antigen-4 (CTLA-4) on blood T-cells during the peripartum period was investigated.

**Results:**

In this study, the incidence of IMIs by any pathogen in early lactation was not associated with a higher expression of PD-1 and CTLA-4 in the periparturient period. However, the incidence of IMIs by major pathogens throughout the first month of lactation was significantly associated with higher expression of PD-1 at 14 days before calving (*P* = 0.03) and CTLA-4 at parturition (*P* = 0.03) by blood T-cells. Also, the expression of CTLA-4 at D0 (*P* = 0.012) by T-cells was associated with the occurrence of persistent IMIs during the first month of lactation.

**Conclusions:**

To our knowledge, this is the first report to investigate the expression of PD-1 and CTLA-4 by blood T-lymphocytes during the periparturient period in dairy cows and to explore their relationship with the incidence of new IMIs in the postpartum period. Thus, a comprehensive understanding of leukocyte biology during peripartum would appear to be a prerequisite for the identification of resilient dairy cows or targets innovative (immunological) non-antibiotic approaches in the transition period.

## Background

The periparturient period in dairy cows is characterized by immunosuppression and marked metabolic changes [1]. During this period, dairy cows are usually in a state of negative energy balance (NEB), which is associated with the mobilization of body fat reserves leading to increased blood non-esterified fatty acids (NEFA) and β-hydroxybutyric acid (BHB) concentrations [2]. This critical period accounts for most of the episodes of new infectious diseases, also mastitis, and metabolic disorders in dairy cows [1–3].

There is a growing interest in specific molecules in immune cells, especially in T-cells, such as programmed cell death protein 1 (PD-1) and cytotoxic T-lymphocyte-associated antigen-4 (CTLA-4). For instance, the success of immune checkpoint inhibition in human cancer therapy suggests that targeting similar pathways might also be useful for preventing and treating infectious diseases [4]. T-cell exhaustion, reflected by the expression of so-called immune checkpoint molecules, is a hallmark of persistent infections as it restrains cell-mediated protective immunity [5]. Indeed, recent studies have demonstrated that immunoinhibitory molecules play a critical role in the immune exhaustion and progression of bovine leukemia viral disease, Johne’s disease and bovine anaplasmosis [6, 7]. Interestingly, immune checkpoint blockade in bovine leukemia virus-infected dairy cows restored the antiviral immunity [8]. Hence, we must explore the role of the checkpoint molecules during the periparturient period, a most critical period of the cow’s life, and their potential associations with the costliest disease in dairy cows, i.e., mastitis, the inflammation of the mammary gland in response to invading bacteria.

Thus, we investigated the expression of PD-1 and CTLA-4 by blood T-lymphocytes during the periparturient period in dairy cows and their relationship with the incidence of new intramammary infections (IMIs) in the postpartum period.

## Results

The bacteriological outcomes of the udder quarters milk samples are presented in the Table 1. Using a generalized logistic regression model, there were no significant association between parity and breed with the occurrence of IMIs by all pathogens or by major mastitis pathogens only, persistent IMIs by all pathogens, or new IMIs by all pathogens or major pathogens only. The serum concentration of BHB, NEFA, and Hp did not show any relationship with the new IMIs.

**Table 1:** Milk bacteriological outcomes of 104 udder quarters analyzed throughout the first month of lactation

Pathogens isolated	D0	D3	D7	D15	D30
Negative	55 (52.88%)	62 (59.62%)	70 (67.31%)	55 (52.88%)	57 (54.81%)
*Staphylococcus chromogenes*	22 (21.15%)	23 (22.12%)	20 (19.23%)	26 (25.00%)	20 (19.23%)
*Staphylococcus aureus*	10 (9.62%)	7 (6.73%)	4 (3.85%)	7 (6.73%)	9 (8.65%)
*Staphylococcus hyicus*	8 (7.69%)	0 (0.00%)	5 (4.81%)	5 (4.81%)	5 (4.81%)
*Corynebacterium ulcerans*	4 (3.85%)	5 (4.81%)	1 (0.96%)	4 (3.85%)	6 (5.77%)
*Staphylococcus cohnii*	2 (1.92%)	1 (0.96%)	0 (0.00%)	0 (0.00%)	0 (0.00%)
*Streptococcus dysgalactiae*	2 (1.93%)	1 (0.96%)	0 (0.00%)	1 (0.96%)	2 (1.92%)
*Rothia endophytica*	1 (0.96%)	0 (0.00%)	0 (0.00%)	0 (0.00%)	0 (0.00%)
*Staphylococcus xylosus*	0 (0.00%)	3 (2.88%)	3 (2.88%)	2 (1.92%)	0 (0.00%)
*Acinetobacter nosocomialis*	0 (0.00%)	1 (0.96%)	0 (0.00%)	0 (0.00%)	0 (0.00%)
*Staphylococcus capitis*	0 (0.00%)	1 (0.96%)	0 (0.00%)	0 (0.00%)	0 (0.00%)
*Staphylococcus epidermidis*	0 (0.00%)	0 (0.00%)	1 (0.96%)	0 (0.00%)	0 (0.00%)
*Pseudomonas stutzeri*	0 (0.00%)	0 (0.00%)	0 (0.00%)	1 (0.96%)	0 (0.00%)
*Streptococcus pluranimalium*	0 (0.00%)	0 (0.00%)	0 (0.00%)	2 (1.92%)	3 (2.88%)
*Escherichia coli*	0 (0.00%)	0 (0.00%)	0 (0.00%)	1 (0.96%)	1 (0.96%)
*Staphylococcus warneri*	0 (0.00%)	0 (0.00%)	0 (0.00%)	0 (0.00%)	1 (0.96%)
*Trueperella pyogenes*	0 (0.00%)	0 (0.00%)	0 (0.00%)	0 (0.00%)	1 (0.96%)
Contaminated	0 (0%)	0 (0%)	0 (0%)	0 (0%)	0 (0%)

D0: day at calving; D3: 3 days after calving; D7: 7 days after calving; D15: 15 days after calving; D30: 30 days after calving

**Fig. 1:**
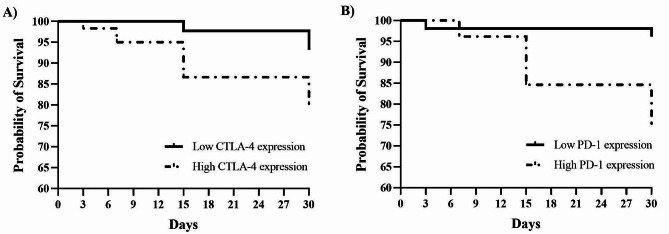
Kaplan-Meier survival curve showing higher incidence of new intramammary infections by major mastitis pathogens in udder quarters in dairy cows during the first month of lactation with high expression (geometric mean fluorescence intensity) of CTLA-4 at parturition (A; *P* = 0.001) and PD-1 at 14 days before the expected day of calving (B; *P* = 0.03) by blood T-lymphocytes. CTLA-4: cytotoxic T-lymphocyte-associated antigen-4; PD-1: programmed cell death protein 1. A drop in survival probability indicates the occurrence of new IMIs (“failure”) by major mastitis pathogens within that specific period

Using the logistic regression model, no effect on the expression of CTLA-4 and PD-1 by T-cells was observed on: (1) occurrence of IMIs by any pathogen (including all pathogens); (2) occurrence of IMIs by major mastitis pathogens only; or on (3) new IMIs by any pathogen (including all pathogens). However, using the logistic regression model, the expression of the immune checkpoints CTLA-4 measured at D0 (*P =* 0.03) and PD-1 measured at D-14 (*P =* 0.03) by T-cells was associated with the occurrence of new IMIs by major pathogens throughout the first month of lactation. Another interesting finding of our study was the association between the expression of CTLA-4 at D0 (*P =* 0.012) by T-cells with the occurrence of persistent IMIs throughout the first month of lactation. Indeed, the Kaplan-Meier survival curve analysis showed that the dairy cows with high expression of CTLA-4 at D0 and PD-1 at D-14 had a higher incidence of new IMIs by major mastitis pathogens through the first month of lactation (Fig. 1).

## Discussion

T-cell exhaustion is characterized by the expression of immune checkpoints, such as CTLA-4 and PD-1, and it is associated with negative regulation of T-cell immunity. Despite the relevance of T-cells for mucosal immunity [9], including for bovine mammary gland [10, 11] and the growing evidence that immunosuppression underpins the susceptibility of periparturient dairy cows to mastitis, this study was the first to investigate the role of immunological checkpoints in bovine mastitis. In agreement, Souza et al. [9] showed that higher expression of CTLA-4 and PD-1 in blood T-lymphocytes at parturition was associated with postpartum uterine health in dairy cow status.

Overall, our results are consistent with the hypothesis that T-cell exhaustion, which may result in weakened effector function, poor recall responses, and a transcriptional state distinct from that of effective effector or memory T-cells, which was associated with limit pathogen clearance [4] resulting in higher susceptibility to infections in dairy cows during the periparturient period and favoring infection persistence.

As a result, as antibodies that block these immune checkpoints have been successfully used to treat and control several types of cancer and even infectious diseases, research into these immune checkpoints has the potential to develop clinical strategies for treating and controlling veterinary infectious diseases by enhancing the host’s immune response to fight diseases [4, 12].

We hypothesized that the low number of dairy cows (*n* = 6, 23.08%) with subclinical ketosis (BHB levels > 0.8 mmol/L) [13] could justify the lack of any association between the serum concentration of BHB and NEFA with the new IMIs. Finally, although the serum concentration of Hp is useful for diagnosing pathological conditions during the periparturient period in dairy cows, its increased levels are not strictly related to mastitis [14], which could explain our results of any association between Hp and the new IMIs.

## Conclusion

In conclusion, our findings highlight the potential value of studying immune checkpoints to identify early dairy cows that are at a higher risk of developing bovine mastitis by major mastitis pathogens during the post-partum period, and also open new paths for the development of novel non-antibiotic approaches for managing bovine mastitis through boosting the host’s immune response.

## Methods

### Animals, samples and experimental design

Twenty-six clinically healthy dairy cows, with no detectable clinical disease at first sampling (seven primiparous and 19 multiparous dairy cows between 2nd and 5th lactations; mean + SEM = 3.26 + 0.21) from two commercial dairy farms (sixteen Guzerá dairy cows at farm A and 10 Girolando dairy cows at farm B) were included. Each of the two dairy farms had 45 lactating dairy cows.

The zebu Guzerá cows were grazed on Massai grass pasture, which is a natural hybrid of Panicum maximum and Panicum infestum, and received concentrates based on their milk production, such as soybean meal, corn meal, and cottonseed meal and cake, along with vitamin and mineral supplements. They were milked once daily by hand with the calf at foot, and produced an average of 20 kg milk per day; the calves were kept with their dams for half of the day due to their high economic values. No pre-dipping and post-dipping, as well dry cow therapy, were performed.

The Girolando dairy cows spent the morning grazing on Mombaça grass (Panicum maximum) pasture and received palm as roughage concentrates based on their milk production, such as soybean meal, corn meal, and cottonseed meal, along with vitamin and mineral supplements. The Girolando dairy cows were milked twice per day by milking machine and produced an average of 27 kg of milk per day. The following mastitis control practices were implemented during milking: forestripping using a strip cup test for clinical mastitis diagnosis; pre-dipping with a solution containing hydrogen peroxide, surfactants, and glycerin (Prima, DeLaval); and drying teats with paper towels. Following milking, post-dipping with iodine (DellaBarrier™, DeLaval) was used. Furthermore, selective dry cow therapy and clinical mastitis treatments were applied. Dairy cows infected by major mastitis pathogens were segregated from the milking line, and milked last.

Blood samples were collected from the 26 aforementioned dairy cows 14 days before the expected day of calving (D-14; mean + SEM = 18.11 + 1.65 days) and at calving (D0) to determine the blood T-lymphocytes expression of CTLA-4 and PD-1. In addition, blood samples were collected at D0, 10 and 30 days after parturition (D10 and D30, respectively) to measure the serum concentrations of BHB, NEFA and haptoglobin (Hp). Milk samples were aseptically collected at D0 and 3 (D3), 7 (D7), 15 (D15), and 30 (D30) days after parturition for microbiological analysis.

### Blood sampling

Peripheral blood samples were aseptically collected by venipuncture of the jugular vein in vacutainer® tubes containing sodium heparin (cat. n. 367,871, BD Biosciences, New Jersey, USA) for flow cytometry analysis, and without anticoagulant (cat. n. 367,812, BD Biosciences, New Jersey, USA) for BHB, NEFA and Hp measurement. Blood samples collected without anticoagulant were centrifuged at 2,500 g for 10 min at room temperature.

### Milk sampling

First, the strip cup test was performed to determine the presence or not of clinical mastitis. Cows showing clinical mastitis symptoms were not included in the study. After discarding the first three milk streams, the teats apices were scrubbed with 70% ethanol using cotton balls and milk samples (*n* = 520 in total) from the individual udder quarters were aseptically collected for microbiological analysis.

### Microbiological analyses

Bacteriological analysis of the milk samples was performed by cultivating 10 µL on 5% defibrinated sheep blood agar plates, which were incubated at 37 °C for 24 to 72 h [15]. The bacterial identification was performed by matrix-assisted laser desorption/ionization time-of-flight mass spectrometry, as previously described [16].

### Definition of IMI, new IMI and persistent IMI

An udder-quarter was defined to be infected (= to have an IMI) if at least 100 CFU mL-1/milk were detected in the milk culturing. A new IMI was defined as a non-infected quarter (= without an IMI) initially that became infected at the subsequent milk sampling [17] or when another pathogen, absent in the previous sampling, was cultured. A persistent IMI was defined when the same pathogen was cultured in the same infected quarter at three consecutive samplings with at least one week interval among samplings [18]. Milk samples yielding > 3 distinct bacterial species were regarded as contaminated. *Staphylococcus aureus*, *Streptococcus* spp., or Gram-negative bacteria were considered major pathogens.

### Serum concentrations of NEFA, BHB, and Hp

The BHB and NEFA serum concentrations were measured using an automated analyzer (Randox Rx Daytona Chemistry AnalyserTM, UK) and Randox® commercial kits (Randox Laboratories, UK) for NEFA (Randox, cat. n. FA115) and BHB (Randox, cat. n. RB 1007). The serum concentrations of the acute-phase protein Hp were determined using a colorimetric technique that detects the production of Hp-haemoglobin complexes, indicating variations in peroxidase activity [9, 19].

### Expression of PD-1 and CTLA-4 in the T-lymphocytes

The expression of PD-1 and CTLA-4 in T-lymphocytes was performed as previously described [9]. Briefly, a Ficoll-PaqueTM PLUS density gradient (GE Healthcare, Germany) was used to isolate peripheral blood mononuclear cells (PBMCs) following the manufacturer’s instructions. Then, PBMCs were incubated at room temperature for 30 min with the primary monoclonal antibodies: mouse anti-bovine IgG1 antibody CD3 (clone MM1A, cat. n. BOV 2009, Washington State University Monoclonal Antibody Center, USA) and goat anti-human CTLA-4 cross-reactive with cattle (cat. n. AF-386-PB, R&D Systems, USA) or goat anti-human PD-1 with cross-reaction with cattle (cat. no. LS-C55247-100, LSBIO, USA). Then, cells were incubated for 30 min at room temperature with the cross-adsorbed donkey IgG conjugated with Alexa Fluor 488 (cat. n. A11055, Thermo Fisher, USA) and goat anti-mouse IgG1-conjugated secondary antibodies conjugated with PE-Texas Red (cat. n. M32017, Thermo Fisher, USA). Finally, the cells were resuspended in 300 µL of PBS with 1% heat-inactivated foetal bovine serum. The samples were analysed with a BD FACSCantoTM II flow cytometer (BD Biosciences, USA). For this study, 10,000 cells were evaluated from each sample. As compensating controls, non-stained control, secondary antibody control, and simple stained PBMC samples were also generated. The data were analyzed using Flow Jo Tree Star software (FlowJo - Treestar 10.5.3 for Windows, Tree Star Inc., USA).

### Statistical analysis

Data distribution was initially assessed using the Shapiro-Wilk test. In this study, the explanatory variables were expression of CLTA-4 and PD-1 by T-lymphocytes at D-14 and D0, respectively, parity (primiparous vs. multiparous) and the breed (Guzerá vs. Girolando), and the dependent variables being (1) occurrence of IMI by any pathogen (including all pathogens); (2) occurrence of new IMI by any pathogen (including all pathogens); (3) occurrence of new IMIs by major mastitis pathogens only; (4) occurrence of IMIs by major mastitis pathogens only; and (5) occurrence of persistent IMIs by any pathogen (including all pathogens). We included in the multivariate logistic model only those variables with *P*-values ≤ 0.20, as established by a univariate logistic model. Then, all variables of interest were analysed until we found a model that provided the greatest biologically relevant explanation for the measured responses. Serum levels of BHB, NEFA and Hp were analysed for their association with dependent variables using an F-test (ANOVA) and Spearman correlation tests. The statistical analyses were performed using InfoStat (Argentina). The statistical models regard the quarters nested within cows and farm, and the cows nested within the farm.

Furthermore, Kaplan-Meier survival curves were created to determine if expression of blood T-cell expression of CLTA-4 and PD-1 at D-14 and D0, respectively, was associated with the development of new IMIs by any pathogen or new IMIs by major mastitis pathogens only through the first month of lactation using InfoStat (Argentina). As there is no reference standard value for CTLA-4 and PD-1 expression by blood T-cells in dairy cows, we used the median value to distinguish between dairy cows with high and low CTLA-4 and PD-1 expression values. The results are expressed as the mean ± standard error, and α = 5% was used for all analyses.

## Data Availability

No datasets were generated or analysed during the current study.

## Abbreviations

PD-1: Programmed cell death protein 1
CTLA-4: Cytotoxic T-lymphocyte-associated antigen-4
BEN: Negative energy balance
NEFA: Non-esterified fatty acids
BHB: β-hydroxybutyric acid
HP: Haptoglobin
IMIs: Intramammary infections
PBMC: Peripheral blood mononuclear cell
PBS: Phosphate-buffered saline
